# An improved approach of salting-out solvent-free microwave mediated rotary distillation for essential oil preparation from fresh leaves of magnolia (*Oyama sieboldii*)

**DOI:** 10.1016/j.fochx.2022.100524

**Published:** 2022-11-26

**Authors:** Xinyu Yang, Ru Zhao, Mengxia Wei, Huiyan Gu, Jialei Li, Lei Yang, Tingting Liu

**Affiliations:** a Key Laboratory of Forest Plant Ecology, Ministry of Education, Northeast Forestry University, Harbin 150040, China; b Key Laboratory of Quality and Safety of Agricultural Products of Nanjing, Nanjing Xiaozhuang University, Nanjing 211171, China; c State Key Laboratory of Esophageal Cancer Prevention & Treatment and Henan Key Laboratory for Esophageal Cancer Research of The First Affiliated Hospital, Zhengzhou University, Zhengzhou 450052, China; d School of Forestry, Northeast Forestry University, Harbin 150040, China; e Food Processing Institute, Heilongjiang Academy of Agricultural Sciences, Harbin 150086, China; f Heilongjiang Provincial Key Laboratory of Ecological Utilization of Forestry-Based Active Substances, Harbin 150040, China; g College of Pharmacy, Qiqihar Medical University, Qiqihar 161006, China

**Keywords:** Salting-out solvent-free microwave mediated rotary distillation (SOSFMRD), *Oyama sieboldii*, Fresh leaves, Essential oil, Magnesium chloride

## Abstract

An improved method denoted as salting out-solvent-free microwave rotary distillation (SOSFMRD) was successfully developed for the extraction of essential oils from fresh magnolia (*Oyama sieboldii*) leaves, in which we achieved the rotation of the reaction material by means of a rotating motor to subject the material to uniform microwave irradiation. Magnesium chloride was selected as the salting-out agent through a comparative study on the salting-out effects of different anions and cations of metal salts. The variables of SOSFMRD were systematically optimized. Under the obtained optimization conditions, the essential oil yield was 21.68 ± 1.02 mL/kgDW. Gas chromatography–mass spectrometry analysis showed that the main chemical constituent of *O. sieboldii* essential oil was dehydrocostuslactone, the content of which reached 30.23 ± 1.27 %. Compared with the other conventional methods, this method has a high yield and low energy consumption, which can effectively reduce impact on the environment.

## Highlights

• Salting-out solvent-free microwave rotary distillation to obtain essential oil.
• MgCl_2_ was selected as a salting-out agent from a wide range of metal salts.
• During the whole process, the material is in a rotating state and is heated evenly.
• Essential oil was prepared from fresh leaves of *Oyama sieboldii.*
• The main component of *O. sieboldii* essential oil were dehydrocostuslactone.

## Introduction

*Oyama sieboldii*, which is also known as *Magnolia sieboldii*, belongs to the Magnoliaceae, a small deciduous tree of the genus *Oyama*. Its leaves are membranous, obovate or broadly obovate, 9–25 cm long and 4–12 cm wide (https://www.iplant.cn/foc). *O. sieboldii* is distributed in a narrow geographical area of the Northern Hemisphere, in China's Jilin, Liaoning, and coastal provinces, and in North Korea and Japan (Chen, Zu, & Yang, 2015). *O. sieboldii* is a very attractive gardening plant and an economically important tree species. It has graceful flowers, elegant colors, and dense foliage. It is often planted as a garden ornamental tree. Furthermore, the leaves of *O. sieboldii* contain essential oil, which can effectively inhibit the production of nitric oxide (NO) and prostaglandin E_2_ (PGE_2_) (Lim et al.,2002), and, by downregulating the expression of tumor necrosis factor (TNF-A), interleukin 6 (IL-6), and interleukin 10 (IL-10), effectively inhibit skin photoaging (Zhang et al., 2021). The essential oil of *O. sieboldii* has a delicate, pleasant scent, and therefore has important application value in the field of cosmetics. In addition, the leaves and bark of *O. sieboldii* contain costunolide (Park et al., 2001) and dehydrocostus lactone (Lee et al., 2014), which have bactericidal effects on *Helicobacter pylori* (Lee et al., 2014, Park et al., 1997) and some important pharmacological effects, such as anti-inflammatory (Oyungerel, Lim, Lee, Choi, Li, & Choi, 2013) and antitumor (Park et al., 2001, Oyungerel et al., 2013) effects. It is also used as wild vegetables for foods and beverages in the Changbai mountain area, China (Liu et al., 2008, Guo et al., 2012.). *O. sieboldii* extracts have received wide attention as raw materials for cosmetics and pharmaceuticals.

At present, hydrodistillation, microwave-assisted hydrodistillation, organic solvent extraction and supercritical CO_2_ extraction are the main methods used to obtain essential oil from plants (Gavahian et al., 2015, Conde-Hernández et al., 2017). Hydrodistillation is a general method in the chemical industry for separating high boiling point or thermally unstable components. Its basic principle is to use the fact that water and organic matter are not mutually soluble and effectively lower the boiling point of organic matter through the partial pressure of water vapor without changing the total operating pressure. Currently, hydrodistillation is commonly used for the separation of essential oils from plants. However, these methods often have some problems, such as long reaction times, high energy consumption, and low yields (Boutekedjiret, Bentahar, Belabbes, Bessiere, 2003). Fortunately, some studies have achieved the so-called salting-out effect by adding certain inorganic salts to the system to enhance the distillation process of essential oils (Yang & Zhang, 2017). It has been shown in the literature that the addition of lithium salts to the conventional hydrodistillation process is effective for improving the yield of essential oils (Li, Zhang, Jie-Xing, & Qin, 2015). Although there have been sporadic publications on increasing the yield of essential oils by salting out with the addition of NaCl or LiCl, there is no systematic study of the anions and cations constituting salt, and there is no way to deeply investigate the mechanism of salting out to improve the yield of essential oils. From the point of view of molecular action, the effect of salt on the vapor–liquid equilibrium is realized by the interaction between the anions and cations in the salt molecule and other components in the system. In aqueous systems, salts can preferentially solvate with certain components in the system through the effects of affinity, hydrogen bonding, and ion electrostatics, and these effects are directly related to the composition of anions and cations. The composition of anions and cations will make it possible to further improve the yield of essential oils and understand the underlying mechanism in this process.

However, even if the salting-out effect is introduced in hydrodistillation, this process still has problems such as high operating temperatures, longer extraction times and high water consumption. Ferhat et al. developed a method combining microwave irradiation with a Clevenger apparatus for essential oil extraction with good results (Ferhat, Tigrine-Kordjani, Chemat, Meklati, & Chemat, 2007). People use the strong penetrability of high-frequency electromagnetic waves to quickly penetrate the plant cell wall, solvent, and other media and reach the interior of the cells. Microwave energy is quickly transformed into heat energy in the plant cells and instantly vaporizes the water in the cells, thereby creating pressure on the cell wall. When the pressure inside the cell exceeds the pressure that the cell wall can withstand, the cell wall is distended, releasing the cellular contents into the surrounding solvent (Mandal et al., 2007, Abdullah et al., 2006). In the same way, the instantaneous high temperature generated by microwave irradiation can also allow the essential oil components in the plant cells to quickly break through the cell wall and be transported by the water vapor (Lucchesi, Chemat, & Smadja, 2004). The yield of plant essential oil separation by microwave-assisted hydrodistillation is 1.07–1.23 times that of traditional hydrodistillation (Li et al., 2018, Chen et al., 2016). The solvent-free microwave-assisted extraction technology based on this method can effectively and fully utilize the original water in fresh plants to obtain essential oils (Li et al., 2018) through the so-called solvent-free microwave extraction. The moisture of the plant material has a large effect on the efficiency of the solvent-free microwave extraction, as the moisture must be above 50 % to ensure the yield and essential oil quality (Liu et al., 2018, Abdelhadi et al., 2015). In the aforementioned solvent-free microwave-assisted extraction of essential oils, the water in the system is not very sufficient since it is contained in the fresh material itself, and the reaction vessel and plant material in the microwave oven cavity are relatively static, which causes uneven microwave irradiation of the plant material in the reaction vessel, as well as uneven mixing of the reflux cooling water with the plant material in the reaction vessel. Moreover, the local temperature is too high to produce the coking phenomenon (Li and Zhu 2019). These characteristics not only result in the contamination of the plant essential oils, leading to the degradation of essential oil quality, but also pose certain safety risks. It is an urgent problem to make the material evenly heated and evenly mix and distribute the reflux cooling water with the material.

The purpose of the study in this paper is to adopt a microwave mediated rotary distillation apparatus and introduce the salting-out effect in the process of solvent-free microwave extraction of *O. sieboldii* essential oil. That is, to produce both a salting-out effect and the rapid absorption of microwave energy for rapid heating by the use of solvent-free microwave extraction to achieve rapid acquisition of essential oil and the use of inorganic salts in fresh plant material. At the same time, the reaction bottle will be rotated in the microwave oven cavity by the rotating shaft, the centripetal force and friction force between the sample and the reaction bottle will make the sample turn in the reaction bottle, and the sample will be heated evenly to avoid coking. The main influencing factors in the salting-out solvent-free microwave mediated rotary distillation (SOSFMRD) process, such as the type and amount of salt, rotational speed, microwave irradiation power and time, and water content of the plant material, were systematically optimized by a single factor combined with the response surface method. This method was compared with a conventional method in terms of the essential oil yield, quality, composition, and environmental impact. This study aims to solve the problems of easy loss of heat-sensitive components and high energy consumption and low efficiency during the distillation process of plant essential oils. Introducing the salting-out effect and the rotating device into the distillation process of plant essential oils effectively ensures the uniform heating of plant materials and the rapid overflow of essential oils, effectively avoiding the loss of heat-sensitive components in essential oils. The concept of green environmental protection is closely combined with the efficient obtaining of high-quality plant essential oils. It is expected to become a new method to replace the existing essential oil extraction method.

## Experimental

### Materials and chemicals

The leaves of *O. sieboldii* were picked from trees in the arboretum of Northeast Forestry University (Harbin, China) in September 2020 and were identified by Professor Gu Huiyan of Northeast Forestry University. The *O. sieboldii* trees are approximately 20 years old, 3 trees are randomly selected as sample trees, and the leaves are evenly picked by hand at approximately 1.5–2.5 m above the ground. After picking, the leaves were placed in a plastic Ziplock bag and sealed in a freezer for frozen storage. The moisture content of the leaves was determined to be 76.61 ± 3.25 %. The metal salts used in the experiments were purchased from Aladdin (Shanghai, China).

### Microwave-mediated rotary distillation apparatus design

The microwave mediated rotary distillation apparatus used is shown in Figure S1 (in Supplementary Material). The apparatus is mainly composed of a microwave irradiation unit, rotating speed console, rotating shaft, reflux condenser and Clevenger-extractor. The microwave irradiation unit was refitted by perforating a microwave oven (Galanz WP700 model, Shunde, China). The microwave oven had a constant irradiation frequency of 2.45 GHz and continuously adjustable power (output power from 120 to 700 W). The side of the microwave oven is perforated, and the perforated part was sealed with a polytetrafluoroethylene sheet to seal the edge of the gap to prevent microwave leakage (Chakarothai, Wake, & Fujii, 2021). This microwave oven was modified by the Engineering Teaching Center of Northeast Forestry University. The rotational speed can be controlled through the console panel for continuous adjustment. A round-bottomed flask was placed in a microwave oven and connected to a Clevenger-type extractor and a reflux condenser after connecting the rotating shaft.

### Salting-out solvent-free microwave mediated rotary distillation process

Fresh *O. sieboldii* leaves were mixed with a certain amount of metal salt, and then a mixing and shearing shredder (Royalstar RS-JR08A, Zhongshan, China) was added for crushing. A certain amount of the above materials was accurately weighed into a 1000 mL round bottom flask, placed in the microwave oven and connected to the entire apparatus. Different rotational speeds were set via the console panel, and the condensed water and distillation apparatus were turned on for different times under different microwave irradiation powers. The water vapor containing the essential oil components was cooled and layered in the graduated tube of the Clevenger extractor. After each set time, the essential oil was collected and placed into a glass sample bottle with a lid, and anhydrous sodium sulfate was added to seal and dehydrate the sample. Dehydrated samples were stored at 4 °C until assayed.

The obtainment of experimental samples with different moisture contents was as follows: After fresh leaves were picked, the moisture contents were 76.61 %, 73.19 %, 69.05 %, 64.28 %, 58.93 and 44.22 % after 0, 2, 4, 6, 8, and 10 h of natural water loss in a cool and ventilated place, respectively.

### Extraction kinetics

To evaluate the effect of microwave irradiation power on the *O. sieboldii* essential oil yield, a first-order kinetic model was used in this study, and the relevant mathematical equations are as follows:

(1) $$\ln\left(Y_{e}-Y_{t}\right)=\ln Y_{e}-kt$$

(2) $$Y_{t}=Y_{e}\left[1-exp(-kt)\right]$$

(3) $$Y=\frac{dy}{dt}=k*Y_{e}exp\left(-kt\right)$$

where *Y_t_* and Ye represent the yield of essential oil at *t* and equilibrium, respectively, *k* is the mass transfer coefficient of the whole process, and *Y* is the essential oil yield at any time.

A total of 200 g of fresh leaf material containing 0.86 mmol/g MgCl_2_ was accurately weighed into a 1000 mL round bottom flask. The rotational speed was set to 72 r/min on the speed control panel. The distillation was carried out for 5, 10, 20, 30, 40, 50 and 60 min at microwave irradiation powers of 230, 385, 540 and 700 W. The essential oils obtained after each distillation were processed in the same manner as previously described.

### Box–Behnken design

The principle of the response surface method is to associate the observed values (dependent variables) with process parameters (independent variables) through statistical methods; usually, a second-order regression equation can be obtained. Using the response surface methodology can allow for a greater focus on the interaction effect between various influencing factors based on single-factor experiments (Sodeifian, Azizi, & Ghoreishi, 2014). According to the results obtained from the preliminary experiments, the microwave irradiation frequency *X_1_*, the addition amount of magnesium chloride *X_2_* and the microwave irradiation time *X_3_* were selected as the parameters to be optimized. In this study, a three-factor three-level Box–Behnken design model was constructed. Seventeen groups were generated to perform sequential random experiments according to specified values, including 5 groups of center point repeat experiments, as shown in Table 1. This can be used to estimate the main effect, interaction, and quadratic effect of three independent variables. The fluctuation ranges of variables *X_1_*, *X_2_* and *X_3_* were set as 385–700 W, 0.64–1.07 mmol/gDW and 20–40 min, respectively, for the complete second-order polynomial equation.

(4) $$Y=\beta 0+\sum_{i=1}^{3}\beta iXi\;\text{+}\;\sum_{i=1}^{3}\beta iiXi^{2}+\sum_{i=1}^{2}\sum_{j=i+1}^{3}\beta ijXiXj+\theta$$

where *Y* is the predicted essential oil yield, *X_i_* and *X_j_* are coding independent parameters, *β_0_* is the model intercept, and *β_i_*, *β_ii_*, and *β_ij_* are the coefficients of linear, quadratic, and interactive interactions, respectively. The validity of this experimental design is verified by subsequent experiments.

**Table 1:** Experimental design matrix to screen for variables that determine the yield of essential oil from fresh leaves of *Oyama sieboldii* and ANOVA results^a^.

Run	BBD experiments^b^				ANOVA								
	*X_1_*	*X_2_*	*X_3_*	*Y*	Source	Sum of squares		Degree of freedom		Mean square		*F*-value	*P*-value
1	385	0.64	30	13.44	Model^b^	133.60		9		14.84		172.66	<0.0001^c^
2	700	0.64	30	19.09	*X_1_*	55.46		1		55.46		645.09	<0.0001^c^
3	385	1.07	30	13.39	*X_2_*	0.06		1		0.06		0.65	0.4482
4	700	1.07	30	18.96	*X_3_*	26.23		1		26.23		305.13	<0.0001^c^
5	385	0.86	20	12.94	*X_1_ X_2_*	0.00		1		0.00		0.01	0.9132
6	700	0.86	20	16.81	*X_1_ X_3_*	1.12		1		1.12		13.02	0.0086^c^
7	385	0.86	40	15.83	*X_2_ X_3_*	0.66		1		0.66		7.70	0.0275^c^
8	700	0.86	40	21.81	*X_1_*^2^	20.60		1		20.60		239.59	<0.0001^c^
9	540	0.64	20	15.14	*X_2_*^2^	16.64		1		16.64		193.55	<0.0001^c^
10	540	1.07	20	15.71	*X_3_*^2^	7.80		1		7.80		90.72	<0.0001^c^
11	540	0.64	40	19.25	Residual	0.60		7		0.09			
12	540	1.07	40	18.19	Lack of fit	0.46		3		0.15		4.25	0.0980
13	540	0.86	30	20.31	Pure error	0.14		4		0.04			
14	540	0.86	30	20.55	Corrected total	134.20		16					
15	540	0.86	30	20.14	Credibility analysis of the regression equations								
16	540	0.86	30	20.59	Index mark	Standard deviation	Mean	Coefficient of variation %	Press	R^2^	Adjust R^2^	Predicted R^2^	Adequacy precision
17	540	0.86	30	20.51	*Y*	0.29	17.80	1.65	7.55	0.9955	0.9897	0.9437	39.52

a The results were obtained with Design Expert 7.0 software.

b *X_1_* is the microwave irradiation power (W), *X_2_* is the amount of magnesium chloride (mmol/gDW), *X_3_* is the microwave irradiation time (min), and *Y* is total yield of essential oil (mL/kgDW).

c Significant at *P* < 0.05.

### Comparison with other reference methods

For hydrodistillation (HD), 200 g of accurately weighed *O. sieboldii* leaves was placed in the shearing shredder, sheared and crushed, transferred to a flask, and then added to a certain amount of deionized water to make the liquid–solid ratio reach 6 mL/g. The flask was placed in a heating mantle and connected to the hydrodistillation apparatus based on the Chinese Pharmacopoeia (National Pharmacopoeia Committee (Ed.), 2020). The sample mixture was treated for 4 h at 450 W.

For microwave hydrodistillation (MHD), 200 g of accurately weighed *O. sieboldii* leaves were put into the shearing shredder, sheared, crushed, and transferred to a flask, and then a certain amount of deionized water was added to achieve a liquid–solid ratio of 6 mL/g. The flask was placed in a microwave oven and connected to the apparatus based on a document (Liu, Zhang, & Yang et al., 2011). The sample mixture was irradiated by a microwave for 40 min at 650 W.

For solvent-free microwave distillation (SFMD), 200 g of accurately weighed *O. sieboldii* leaves were placed in a shearing shredder, sheared, crushed, and transferred to a flask, which was placed in a microwave oven and connected based on references (Peng et al., 2021, Ma et al., 2012). The essential oil was distilled for 40 h at 650 W.

### Gas chromatography–mass spectrometry analysis of essential oil

An Agilent 7890B/7000C (Agilent Technology Co., Ltd., Santa Clara, CA, USA) gas chromatography-mass spectrometer was used for the essential oil component analysis in this study. The instrument was equipped with an HP-5MS column (Agilent, 30 mm × 0.25 mm, 0.25 μm film thickness) and a mass spectrometer (70 eV) operating in electron ionization mode. The GC–MS operating conditions were slightly modified from reference (Chen et al., 2015) as follows: the injection volume was 1 µL; the helium flow rate was 1.5 mL/min; the split ratio was 1:80; the inlet temperature was 250 °C; and the detector temperature was set to 280 °C. The heating program was as follows: the initial temperature was 50 °C and held for 5 min, then increased to 260 °C at a rate of 5 °C/min and held for 5 min, and finally increased to 280 °C at an incremental rate of 10 °C/min. The energy of the EI ion source was 70 eV. The atomic weight scan ranged from 15 to 500 *m*/*z*. Mass spectra determined by GC–MS were compared with those of the National Institute of Science and Technology (NIST14) library to determine the constituents of the essential oil.

## Results and discussion

### Single-factor experiments

#### Screening of salts

**Fig. 1:**
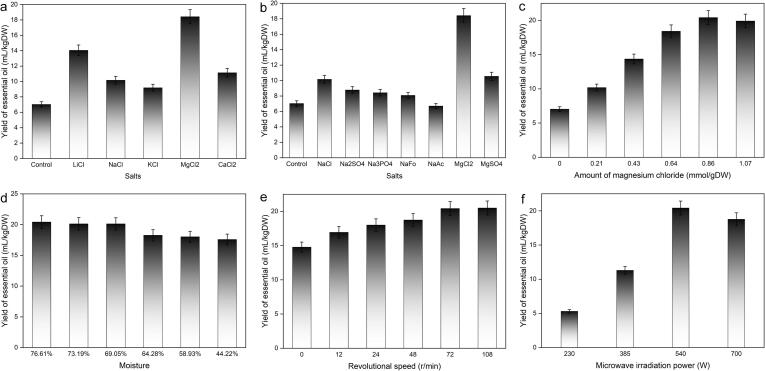
Effect of (a) cation, (b) anion, (c) amount of magnesium chloride, moisture content (d), rotational speed (e), and microwave irradiation power (f) on the yield of essential oil from fresh leaves of *Oyama sieboldii*.

The anion and cation compositions of a salt has a significant effect on the methods in this experiment, which may greatly affect the target analyte yield. In this work, the effect of the compositions of anions and cations was investigated, and the same molar ratio was used as the evaluation standard for different salt systems. Different kinds of salts (0.16 mol) were added to 200 g of fresh leaves of *O. sieboldii*, and solvent-free rotary microwave extraction was carried out under a microwave irradiation power of 540 W. The moisture content of the material was 76.61 %, and the rotational speed of the reaction flask was 72 r/min. The results are shown in Fig. 1. In Fig. 1a, the effects of five inorganic salts with the same anion (Cl^-^) but different cations on the yield of essential oil were compared. Among them, LiCl, NaCl and KCl are all chloride salts of alkali metal ions of the first main group of the periodic table of elements. Their effect on the yield of *O. sieboldii* essential oil decreases in order, which means that when the number of outer electrons is the same, then the smaller the cation radius contained in the salting-out agent is, the greater the influence on the hydration layer of the ions, the stronger the ion dehydration effect, and the greater the salting-out effect. In Fig. 1a, the salting-out effect of alkaline earth metal salts Mg^2+^ and Ca^2+^ under the same Cl^-^ is also compared. Similar to the alkali metal salts, the salting-out effect of Mg^2+^ in the third cycle is better than that in the fourth cycle of Ca^2+^, which verifies the relationship between the salting-out effect and the ionic radius. When comparing Na^+^ and Mg^2+^, and K^+^ and Ca^2+^ in the same cycle, the salting-out effect of the higher-valence ions is stronger; this result is consistent with the experimental results of the salting-out effect in the extraction of phenolic and carboxylic acid compounds found in the literature (Asrami and Saien, 2018, Tu et al., 2017). The distillation operations of all the added salts in this paper showed the salting-out effect, and the yields of essential oils were higher than those of the control samples without salts, which confirmed that the addition of metal salts during the distillation process can indeed improve the yield of essential oils (Yan et al., 2021, Ai et al., 2016). When the cation is the same (Na^+^) but the anion is different, as shown in Fig. 1b, which compares the salting-out effects of NaCl, Na_2_SO_4_ and Na_3_PO_4_, the salting-out effect of NaCl is the best, followed by Na_2_SO_4,_ then Na_3_PO_4_, and the salting-out effect of MgCl_2_ is obviously better than that of MgSO_4_, which indicates that in the case of the same cation, the lower the valence of the anion is, the weaker the salting-out effect. However, the differences in the salting-out effects are smaller than those between the cation groups. This is because water has a strong solvation effect on both anions and cations, but it has a stronger solvation effect on cations than on anions, so the salting-out effect is mainly manifested with the solvation of cations. In the two sulfates, Na_2_SO_4_ and MgSO_4_, the salting-out effect of Mg^2+^ ions is greater than that of Na^+^ ions, which is consistent with the effect of chloride salts, again confirming that the salting-out effect of high-valent metal ions is significant. We also added formate (Fo) and acetate (Ac) to the salting-out effect comparison experiments. The results show that the general trend in the salting-out effects is NaCl > Na_2_SO_4_ > Na_3_PO_4_ > NaFo > NaAc. This may explain the relationship between the salting-out effect and the size of the anionic groups, i.e., larger anionic groups lead to a decrease in the salting-out effect. In summary, MgCl_2_ obviously helped to improve the yield of *O. sieboldii* essential oil. MgCl_2_ is a low-cost metal salt that can be used as a food additive, so MgCl_2_ was selected as the salting-out agent for extracting the essential oil of the *O. sieboldii* leaves.

#### Effect of the amount of magnesium chloride

The amount of the salting-out agent magnesium chloride is also a key factor for the optimization of the salting-out effect. The experiments were carried out under the conditions of a microwave irradiation power of 540 W, a rotational speed of 72 r/min, and a microwave irradiation time of 30 min. The effect of the amount of MgCl_2_ on the yield of essential oils in the SOSFMRD process is shown in Fig. 1c. When the amount of MgCl_2_ was increased from 0.21 mmol/gDW to 0.64 mmol/gDW, the yield of essential oils increased significantly, and when the addition of MgCl_2_ was 0.86 mmol/gDW, the yield of essential oil tended to be the largest. Upon further increasing the dosage of magnesium chloride, the yield of essential oil no longer significantly increased. Considering multiple factors, such as energy savings and environmental protection, the range of 0.64–1.07 mmol/gDW was selected for subsequent optimization experiments.

#### Effect of the moisture of the material

The SOSFMRD process is carried out at atmospheric pressure based on a microwave-heated steam distillation process. The *in situ* water inside the plant material rapidly expands and gasifies after being irradiated by microwaves, which ruptures oil-bearing cells and glands. Thus, the process releases the essential oil entrained by the *in situ* water of the plant material by azeotropic distillation (Ma et al., 2012, Lucchesi et al., 2004). The moisture of the plant material is an important factor in this process. The collected fresh leaves of *O. sieboldii* were placed in a cool and ventilated place for varying amounts of time, and 6 groups of fresh leaf samples of *O. sieboldii* with different moisture contents were obtained. The experiments were carried out under the conditions of a microwave power of 540 W, a rotational speed of 72 r/min, a microwave irradiation time of 30 min, and a salt addition of 0.86 mmol/gDW. The effect of leaf moisture on essential oil yield is shown in Fig. 1d. The essential oil yield decreased slightly when the moisture of leaves decreased from 76.61 % to 69.05 %, but when the moisture of leaves decreased from 69.5 % to 64.28 %, the yield of *O. sieboldii* essential oil was significantly reduced. As the moisture of leaves further decreased from 64.28 % to 44.22 %, the yield of essential oil still slowly decreased. There was a difference of 2.84 mL/kgDW between the maximum and minimum yields of essential oils throughout the experiment. This shows that as long as the freshness of the raw materials is guaranteed and the raw materials are not excessively dehydrated, the yield of essential oils can be maintained at a high level. From the perspective of energy savings and environmental protection, it is an advantage that no external solvent is added, which not only saves solvent resources but also avoids the power loss and wastewater pressure treatment required by solvent recovery.

#### Effect of the rotational speed

We selected the newly picked leaves of *O. sieboldii* as the raw material (moisture content 76.61 %). Under the conditions of a microwave irradiation power of 540 W, a microwave irradiation time of 30 min, and an addition of MgCl_2_ of 0.86 mmol/gDW, distillation was carried out at rotational speeds of 0, 12, 24, 42, 72, and 102 r/min. The obtained results are shown in Fig. 1e. The yield of essential oil increases with increasing rotational speed, and the rotation of the materials can indeed significantly improve the yield of essential oils. The yield increased from 14.77 ± 0.61 mL/kgDW in the quiescent state (0 r/min) to 20.42 ± 0.89 mL/kgDW at 72 r/min. However, when the rotational speed was further increased from 72 r/min to 108 r/min, the yield of essential oil did not increase significantly. The material is turned up and down in the flask under the action of the rotating force, which ensures that the plant material absorbs the microwave energy more fully, and the material is heated evenly. However, a rotational speed that is too fast will not only lead to a waste of energy but also increase the risk of danger during operation. Therefore, considering the above factors, we choose 72 r/min as the appropriate rotational speed.

#### Effect of the microwave irradiation power

High-frequency electromagnetic waves can quickly penetrate the interior of the material and transform into heat energy, which rapidly increases the temperature in plant cells. Currently, the water in the plant cells rapidly vaporizes and puts pressure on the cell walls. When the pressure limit of the cell wall is exceeded, the cell ruptures, and the essential oil components are released (Zhao, Yang, Zhang, Zhou, Zhou, & Yang, 2022). Therefore, microwave irradiation power is one of the important factors affecting the yield of essential oil. Under the condition that the addition amount of MgCl_2_ was 0.86 mmol/gDW and the rotational speed was 72 r/min, different microwave irradiation powers (230, 385, 540 and 700 W) were used for distillation for 30 min. It can be seen in Fig. 1f that with the increase in microwave irradiation power through this range, the yield of *O. sieboldii* essential oil showed a trend of first increasing and then decreasing. With an increase in microwave irradiation power from 230 W to 540 W, the yield of essential oil was significantly improved. However, when the microwave irradiation power was further increased from 540 W to 700 W, the yield of essential oils decreased. This may be due to the excessively high microwave irradiation power causing the temperature in plant cells to rise too quickly and the plant material to gelatinize, resulting in a decrease in the yield of essential oils. We chose a range of 385–700 W of microwave irradiation power for further optimization.

#### Effect of the microwave irradiation time and kinetics

**Fig. 2:**
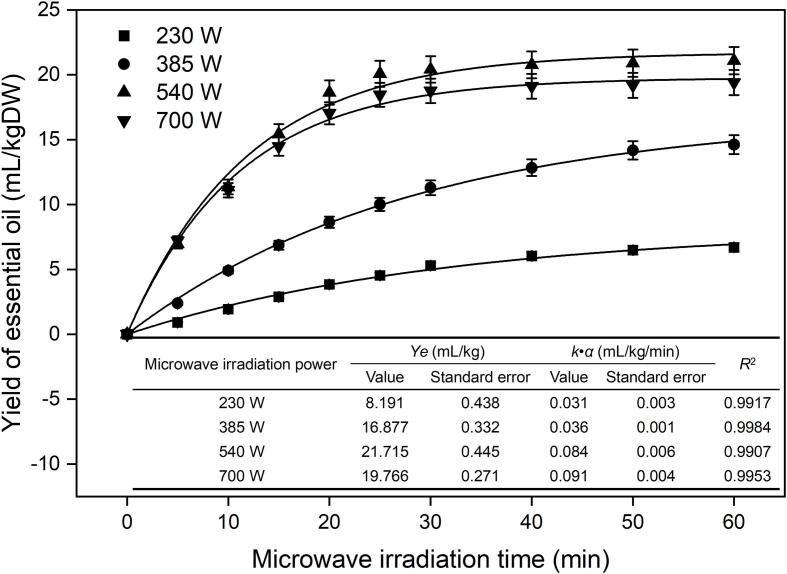
Kinetic curves for yield of essential oil isolated under various microwave irradiation powers.

Fig. 2 shows that the *R*^2^ values under different microwave radiation powers are all greater than 0.99, which indicates that the first-order kinetic model can reflect the actual experimental results very well. Under different microwave irradiation powers (230, 285, 540 and 700 W), the trend of essential oil yield with microwave irradiation time was different. It can be seen that the yield of essential oils increased significantly within the first 20 min for both 540 W and 700 W microwave irradiation power, and then the yield of essential oil did not change much with a further increase in time. Comparing the yield of essential oil after reaching equilibrium, it was found that the yield of essential oil under 540 W microwave irradiation power was higher than that under 700 W microwave irradiation power. Under the microwave irradiation power of 385 W, the yield of *O. sieboldii* essential oil increased obviously in the first 30 min, but overall showed a slow upward trend. Under the condition of 230 W microwave irradiation power, a similar trend can be observed as that of the 385 W microwave irradiation power condition, but the yield of essential oil is always much lower than that of 385 W. Hence, we chose 20–40 min of microwave irradiation time for further optimization.

### Parameter optimization by response surface methodology

To further study the effect of the interaction of the aforementioned factors on the *O. sieboldii* essential oil yield, we next carried out a Box–Behnken design optimization. Based on the results of the above single-factor experiments, three factors, microwave irradiation power (*X_1_*), the amount of MgCl_2_ added (*X_2_*), and microwave irradiation time (*X_3_*), were selected as response independent variables to design subsequent experiments. The yield (*Y*) of *O. sieboldii* essential oil was used as the response value of this experimental design. Table 1 presents the experimental design matrix used to screen for variables that determine the yield of essential oil from the fresh leaves of *O. sieboldii* and analyze the variance results. The correlation coefficient *R^2^* of the essential oil yield model of *O. sieboldii* was 0.9955, which indicated that the model could be used to explain all the variability without obvious underfitting. The value of the adjusted *R^2^* is 0.9897, which is very close to *R^2^*, and the values are all above 0.99, which shows that the obtained model has high accuracy and that the experimental data have a high correlation with the predicted value (Swamy, Sangamithra, & Chandrasekar, 2014). Combining the *F* value of 4.25 and the *P* value of 0.098 (*P* > 0.05), it can be concluded that the obtained model can reasonably explain the experimental data and predict the *O. sieboldii* essential oil yield. The coefficient of the variation value is mainly used to detect the repeatability of the equation (Zhang, Liu, Li, & Chi, 2014). Generally, a value of the coefficient of variation of <10 can prove that the model has good repeatability. In this experiment, the coefficient of variation (CV) is 1.65, which shows that the model can provide high reliability and fitting accuracy. An adequate precision of 39.52 (>4) indicates a sufficient signal. The following quadratic regression equation is obtained:

(5) $$\begin{array}{c} Y=-6.3\times 10^{1}+1.0\times 10^{-1}X_{1}+7.9\times 10^{1}X_{2}+9.8\times 10^{-1}X_{3}-4.9\times 10^{-4}X_{1}X_{2}\\ \;\;\;\;\;\;\;+3.4\times 10^{-4}X_{1}X_{3}-1.9\times 10^{-1}X_{2}X_{3}-8.9\times 10^{-5}X_{1}^{2}-4.3\times 10^{1}X_{2}^{2}-1.4\times 10^{-2}X_{3}^{2} \end{array}$$

**Fig. 3:**
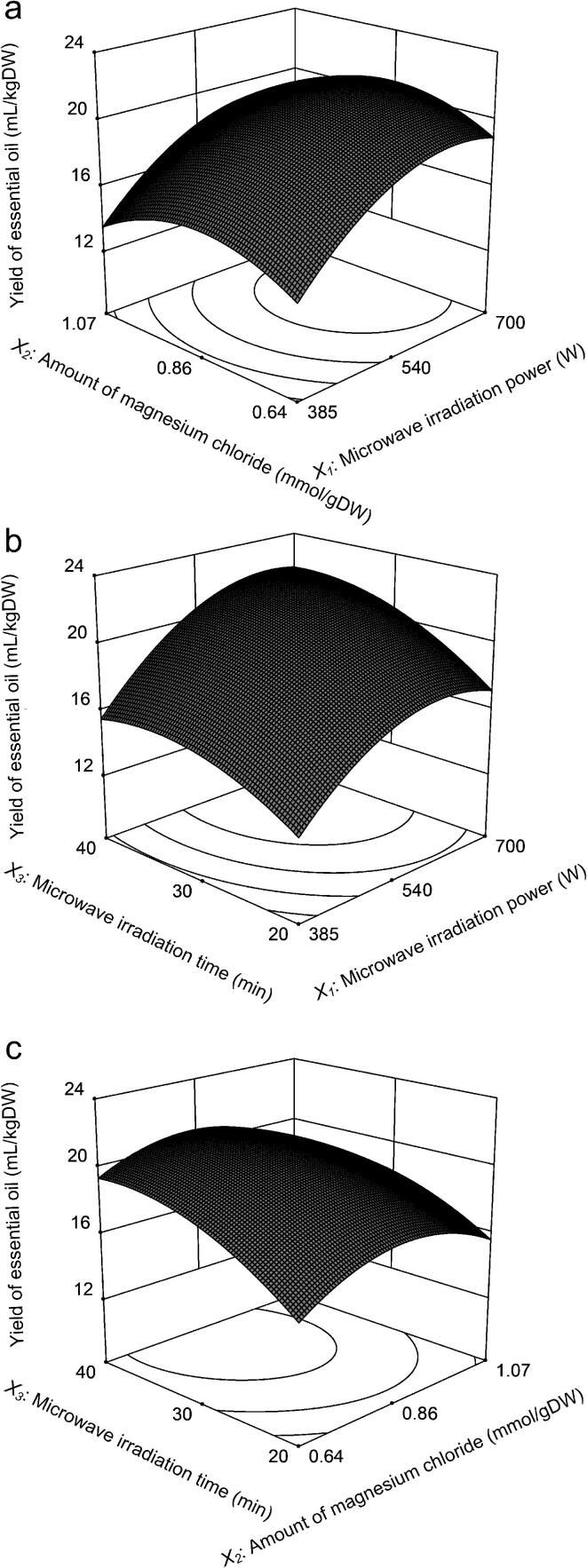
Optimization of essential oil yields by the Box-Behnken design (BBD) method. (a) Interaction of microwave irradiation power and amount of magnesium chloride, (b) interaction of microwave irradiation power and microwave irradiation time, (c) interaction of microwave irradiation time and amount of magnesium chloride.

The 3D response surfaces in Fig. 3 clearly and logically show the effect of each variable on the yield of *O. sieboldii* essential oil. Table 1 shows that the interaction terms *X_1_X_3_* and *X_2_X_3_* have a significant impact on the yield of *O. sieboldii* essential oil (*P* < 0.05) (Chen et al., 2014, Lopresto et al., 2014). The quadratic term *X_1_X_2_* had no substantial effect (*P* greater than 0.05). The optimal experimental parameters calculated by the Box–Behnken design are as follows: the microwave irradiation power is 652 W, the amount of magnesium chloride added is 0.83 mmol/gDW, and the microwave irradiation time is 38 min. Under these conditions, the predicted yield of *O. sieboldii* essential oil is 22 mL/kgDW.

### Verification experiment

We used the optimal experimental conditions obtained after response surface optimization (microwave irradiation power of 650 W, magnesium chloride addition of 0.83 mmol/gDW, and microwave irradiation time of 38 min) to conduct verification experiments. Through three parallel experiments, it was found that the actual yield of *O. sieboldii* essential oil was 21.68 ± 1.02 mL/kgDW under the experimental conditions, which was close to the predicted value of 22 mL/kgDW. The experimental results verify that the optimal conditions obtained by the optimization calculations are reliable and that their experimental implementation provides results that are highly consistent with the results expected by the model.

### Comparison with other reference methods

Different distillation methods not only have a great influence on the yield of essential oils, but the composition of *O. sieboldii* essential oils obtained by the different distillation methods may also be different. The proposed SOSFMRD in this experiment was compared with traditional SOSFMD (salting-out solvent-free microwave distillation), SFMD (solvent-free microwave distillation), MHD (microwave hydrodistillation) and HD (hydrodistillation) in terms of yield, essential oil composition, and environmental impact. The results are as follows.

#### Chemical composition of essential oils

Table 2 contains the components of the essential oils obtained by the five different distillation methods, and a total of 42 components were detected. The identified chemical constituents are also listed in the table with their retention index, molecular formula, and relative percentage. The relative content of the individual components is expressed as a percentage of their peak area relative to the total peak area (RA%). The components in the essential oils obtained by the five distillation methods were all detected and analyzed, accounting for 87 %-96.14 % of the total content. The components in the essential oils obtained by the five methods such as dehydrocostuslactone, β-selinene, β-elemene, β-phellandrene and τ-muurolol accounted for more than 50 % of the peak area. Regardless of the kind of essential oil obtained, the relative content of dehydrocostuslactone was the highest. The relative content of the individual components depends on the method used. The RA% values of dehydrocostuslactone in the essential oils obtained by SOSFMRD, SOSFMD, SFMD, MHD and HD corresponded to 30.23 %, 25.93 %, 32.85 %, 21.13 % and 23.35 %, respectively. Up to 36 components were detected in the essential oil obtained by the SOSFMRD, which was the largest amount of components, followed by the SOSFMD with 32 components. In contrast, only 28 components were detected by the SFMD. The least amount of components was found for MHD and HD, both with only 27 components. The content of eremanthin in the essential oil obtained by SOSFMRD was the highest, which was 6.71 %. The essential oil obtained by SOSFMD contains 0.36 % eremanthin, while the essential oil obtained by the other three methods contains almost no eremanthin. In contrast, HD has the advantage of obtaining *O. sieboldii* essential oil containing more β-elemene than the other methods. Compared with SOSFMRD (1.87 %), SOSFMD (3.56 %), SFMD (3.26 %), and MHD (2.87 %), the essential oil obtained by HD contains the highest amount of β-elemene (8.48 %). This may be because the essential oil obtained by hydrodistillation has fewer components, and thus the relative contents of each of the main components will increase. The content of β-copaene in the essential oils obtained by the four methods except SOSFMRD was approximately 6 %, while the β-copaene content in the essential oil obtained by SOSFMRD was only 1.87 %. Similarly, the β-selinene content of the essential oil obtained by the SFMD was the highest at 10.92 %, while the essential oil obtained by the SOSFMRD contained only 3.45 % β-selinene. For some other components, such as caryophyllene, bicylogermacrene, δ-cadinene and thujopsenal, the relative contents obtained by the five methods compared in this paper showed little difference. The contents of total terpenes in *O. sieboldii* essential oil obtained by SOSFMRD, SOSFMD, SFMD, MHD and HD were 26.67 %, 45.14 %, 43.07 %, 35.28 % and 38.55 %, respectively. The contents of total oxygenated terpenes were 54.80 %, 46.17 %, 53.07 %, 56.78 % and 51.68 %, respectively. Only nonterpene aliphatic and aromatic compounds were detected in the essential oil obtained by SOSFMRD. The above results show that the method used in this study can indeed enrich the essential oil components to a certain extent and can greatly improve the yield of essential oil. However, the effect on the relative content of essential oils was small, and the results were similar to those obtained in the literature (Chen et al., 2015, Chen et al., 2018).

**Table 2:** Gas chromatography–mass spectrometry result for the chemical compositions of essential oil from fresh leaves of *Oyama sieboldii*.

No.^a^	Compounds	RI^b^	ID	Molecular formula	RA^e^(%)				
					SOSFMRD^f^	SOSFMD	SFMD	MHD	HD
1	3-Furfural	831	RIc, MS^d^	C5H4O2	0.85	nd^g^	nd	nd	nd
2	Acetylfuran	904	RI, MS	C6H6O2	0.50	nd	nd	nd	nd
3	5-MethyIfurfural	970	RI, MS	C6H6O2	2.85	nd	nd	nd	nd
4	β-Phellandrene	1007	RI, MS	C10H16	1.81	5.13	3.41	1.55	0.72
5	α-Terpinene	1016	RI, MS	C10H16	0.52	nd	nd	nd	nd
6	β-Cymene	1022	RI, MS	C10H14	0.31	nd	nd	nd	nd
7	Limonene	1030	RI, MS	C10H16	0.57	0.86	0.55	0.25	nd
8	3,6,6-trimethyl-Bicyclo[3.1.1]hept-2-ene		MS	C10H16	3.92	5.34	3.84	1.69	0.97
9	Cyclofenchene	1037	RI, MS	C10H16	0.42	0.62	0.35	nd	nd
10	γ-Terpinene	1056	RI, MS	C10H16	0.71	nd	nd	nd	nd
11	4-Terpinenol	1180	RI, MS	C10H18O	0.83	0.29	nd	0.22	nd
12	Geraniol	1254	RI, MS	C10H18O	nd	nd	0.39	0.43	nd
13	Eugenol	1359	RI, MS	C10H12O2	1.24	nd	nd	nd	nd
14	E)-3,7-Dimethylocta-2,6-dienyl ethyl carbonate		MS	C13H22O3	nd	0.90	nd	0.84	0.36
15	Guaia-10(14),11-diene		MS	C15H24	nd	0.30	nd	nd	0.39
16	β-Elemene	1380	RI, MS	C15H24	1.87	3.56	3.26	2.87	8.48
17	Caryophyllene	1415	RI, MS	C15H24	0.78	1.65	1.51	1.00	1.79
18	α-Guaiene	1426	RI, MS	C15H24	nd	0.58	0.33	0.22	0.51
19	cis,cis,*cis*-1,1,4,8-Tetramethyl-4,7,10-cycloundecatriene		MS	C15H24	0.98	2.18	1.81	1.42	2.01
20	β-Copaene	1430	RI, MS	C15H24	1.87	6.61	6.11	6.50	6.35
21	Humulene	1434	RI, MS	C15H24	1.48	0.82	0.51	nd	nd
22	*cis*-α-Bergamotene	1435	RI, MS	C15H24	3.27	1.33	4.74	3.56	1.72
23	Bicylogermacrene	1467	RI, MS	C15H24	0.50	2.57	1.28	1.22	0.41
24	Guaia-10(14),11-diene		MS	C15H24	nd	0.87	0.86	0.73	0.37
25	β-Selinene	1485	RI, MS	C15H24	3.45	9.45	10.92	9.71	8.78
26	1,1,4,5,6-Pentamethyl-2,3-dihydro-1H-indene	1522.6	RI, MS	C14H20	0.85	nd	nd	nd	nd
27	γ-Cadinene	1524	RI, MS	C15H24	nd	0.33	0.35	nd	0.24
28	δ-Cadinene	1530	RI, MS	C15H24	2.14	2.03	2.58	2.78	3.39
29	α-Nerolidol	1551	RI, MS	C15H26O	0.38	0.45	1.37	2.04	0.61
30	Germacren D-4-ol	1569	RI, MS	C15H26O	0.69	3.71	4.77	8.48	4.60
31	τ-Cadinol	1639	RI, MS	C15H26O	1.00	1.51	1.96	3.63	2.78
32	τ-Muurolol	1645	RI, MS	C15H26O	3.59	2.84	2.49	4.36	3.89
33	Eremophilone	1756.4	RI, MS	C15H22O	2.48	3.82	3.80	5.18	4.47
34	Thujopsenal		MS	C15H22O	0.31	0.38	0.50	0.85	0.63
35	Longifolenaldehyde		MS	C15H24O	0.82	3.11	1.90	4.02	6.19
36	Neophytadiene	1806	RI, MS	C20H38	2.38	0.91	0.67	1.77	2.42
37	Dehydrosaussurea lactone	1838.4	RI, MS	C15H20O2	1.44	1.18	0.80	nd	0.69
38	Dihydrodehydrocostus lactone	1953.9	RI, MS	C15H20O2	2.42	0.52	nd	0.22	0.29
39	Eremanthin		MS	C15H18O2	6.71	0.36	nd	nd	nd
40	Eudesma-5,11(13)-dien-8,12-olide		MS	C15H20O2	2.22	nd	nd	nd	nd
41	Dehydrocostuslactone	2006.7	RI, MS	C15H18O2	30.23	25.93	32.85	21.13	23.35
42	Phytol	2105	RI, MS	C20H40O	0.82	1.17	2.23	5.39	3.81
	Total identified compounds				87.21	91.31	96.14	92.06	90.23
	Total terpene hydrocarbons				26.67	45.14	43.07	35.28	38.55
	Total oxygenated terpenes				54.80	46.17	53.07	56.78	51.68
	Non-terpene aliphatics				3.36	0.00	0.00	0.00	0.00
	Total aromatics				2.39	0.00	0.00	0.00	0.00

a Compounds listed in order of elution from HP-5MS capillary column.

b Retention indices relative to C11–C21 *n*-alkanes on HP-5MS capillary column.

c Tentative identification by comparison with RI on HP-5MS capillary column with literature data.

d Confirmed by comparison with mass data obtained from NIST02 mass spectra library.

e Relative area percentage (peak area relative to the total peak area, %).

f SOSFMRD: salting-out solvent-free microwave rotary distillation; SOSFMD: salting-out solvent-free microwave distillation; SFMD: solvent-free microwave distillation; MHD: microwave hydrodistillation; HD: hydrodistillation.

g Not detected.

#### Environmental impact and economic effect

Global warming is the change in the Earth's climate caused by the development of human society. With the increase in human activity, the “culprit” gas CO_2_ that causes global warming is also increasing. Currently, global warming is affecting people's way of life and causing an increasing number of problems. In recent years, the concept of green chemistry has been advocated worldwide. In this study, carbon dioxide emissions are used as an indicator to measure the impact of the reported method on the environment. The amount of CO_2_ emitted to the atmosphere is calculated according to the literature: for every 1 kW h obtained from coal or other fossil fuels, 800 g of CO_2_ is emitted into the atmosphere during its combustion (Chen et al., 2018). The CO_2_ emission loads of SOSFMRD, SOSFMD, SFME, MHD and HD are listed in Table 3. It can be seen that on the basis of ensuring high essential oil yield, the energy consumption time is <1/5 of the traditional HD method. The emission of CO_2_, the main gas causing the greenhouse effect, was reduced from 1200 g to 342 g. Compared with the SOSFMD, the CO_2_ emission of the SOSFMRD was slightly higher due to the increase in the process of motor rotation. However, the latter has a higher essential oil yield and a richer essential oil composition. In addition, there is a certain difference in power consumption between different methods. This is a measure of the future economic benefits of the method after it is put into production. It can be seen that the SOSFMRD has a lower power consumption. To take into account the essential oil yield as another important indicator for judging economic benefits, this study calculated the essential oil yield per kWh, which is also reported in Table 3. The yield of essential oil per kilowatt hour of SOSFMRD is much higher than that of other methods, even more than 5 times higher than that of traditional HD. It can be shown that this method does obtain more essential oil under the same power consumption. The shorter response time can effectively shorten the working hours of workers, thereby saving labor costs. Considering the above points comprehensively, the further development of this method can result in economic benefits.

**Table 3:** Comparison of environmental impact and economic effect with different methods, each experiment was repeated for three times.

	SOSFMRD^a^		SOSFMD	SFME	MHD	HD
Power-consuming apparatus	Microwave oven	Rotating motor	Microwave oven	Microwave oven	Microwave oven	Heating jacket
Electric power (W)	650	25	650	700	700	500
Energy consumption time (min)	38		38	38	38	180
Electricity consumption (kW h)	0.43		0.41	0.44	0.44	1.5
Yield of essential oil (mean ± SD, mL/kgDW)	21.68 ± 1.02		20.23 ± 0.85	18.34 ± 0.79	17.55 ± 0.81	15.64 ± 0.61
Yield of essential oil per kilowatt hour (mean ± SD, mL/kgDW/kWh)	50.42 ± 2.37		49.34 ± 2.07	41.68 ± 1.80	39.89 ± 1.84	10.43 ± 0.41
Environmental impact (g CO_2_ rejected)	342.00		329.33	354.67	354.67	1200

a SOSFMRD: salting-out solvent-free microwave rotary distillation; SOSFMD: salting-out solvent-free microwave distillation; SFMD: solvent-free microwave distillation; MHD: microwave hydrodistillation; HD: hydrodistillation.

## Conclusion

In this study, SOSFMRD was successfully used to obtain essential oil from fresh *O. sieboldii* leaves. MgCl_2_ was selected as the salting-out agent, and the process parameters optimized by a single factor combined with Box–Behnken design were as follows: a MgCl_2_ addition amount of 0.83 mmol/gDW, a rotational speed of 72 r/min, a microwave irradiation power of 650 W, and a microwave irradiation time of 38 min. The yield of essential oil was 21.68 ± 1.02 mL/kgDW. Analysis by GC–MS showed that the main chemical constituent of *O. sieboldii* essential oil was dehydrocostuslactone. Compared with the essential oils obtained by other conventional methods, the yield of this method was higher, but the composition was similar. In addition, the analysis results of CO_2_ emissions and power consumption show that the SOSFMRD can effectively reduce the impact on the environment. The proposed method shows great promise in the field of essential oil extraction from other plant materials.

## CRediT authorship contribution statement

**Xinyu Yang:** Formal analysis, Investigation, Writing – original draft, Writing – review & editing. **Ru Zhao:** Formal analysis, Validation, Writing – original draft, Writing – review & editing, Methodology. **Mengxia Wei:** Formal analysis. **Huiyan Gu:** Conceptualization, Writing – review & editing. **Jialei Li:** Investigation, Validation. **Lei Yang:** Conceptualization, Writing – review & editing, Funding acquisition. **Tingting Liu:** Validation, Funding acquisition.

## Declaration of Competing Interest

The authors declare that they have no known competing financial interests or personal relationships that could have appeared to influence the work reported in this paper.

## Data Availability

Data will be made available on request.
