# Supplementary material for: An improved approach of salting-out solvent-free microwave mediated rotary distillation for essential oil preparation from fresh leaves of magnolia (Oyama sieboldii)
Source: Food Chem X. 2022 Nov 26;16:100524. doi: 10.1016/j.fochx.2022.100524 (PMC9743287; doi:10.1016/j.fochx.2022.100524)
Supplement: Supplementary data 1 [file mmc1.docx]

Supplementary material

# An improved approach of salting-out solvent-free microwave mediated rotary distillation for essential oil preparation from fresh leaves of magnolia (*Oyama sieboldii*)

Xinyu Yang^a†^, Ru Zhao^b†^, Mengxia Wei^a,c^, Huiyan Gu^d^, Jialei Li^e^, Lei Yang^a,f^*, Tingting Liu^g,^*

^a^ Key Laboratory of Forest Plant Ecology, Ministry of Education, Northeast Forestry University, Harbin 150040, China

^b^ Key Laboratory of Quality and Safety of Agricultural Products of Nanjing, Nanjing Xiaozhuang University, Nanjing 211171, China

^c^ State Key Laboratory of Esophageal Cancer Prevention & Treatment and Henan Key Laboratory for Esophageal Cancer Research of The First Affiliated Hospital, Zhengzhou University, Zhengzhou 450052, China

^d^ School of Forestry, Northeast Forestry University, Harbin 150040, China

^e^ Food Processing Institute, Heilongjiang Academy of Agricultural Sciences, Harbin 150086,

China

^f^ Heilongjiang Provincial Key Laboratory of Ecological Utilization of Forestry-Based Active Substances, Harbin 150040, China

^g^ College of Pharmacy, Qiqihar Medical University, Qiqihar 161006, China

^†^ These authors contributed equally to this work.

* Corresponding author E-mail: yanglei@nefu.edu.cn (L. Yang); ltting@outlook.com (T. Liu)


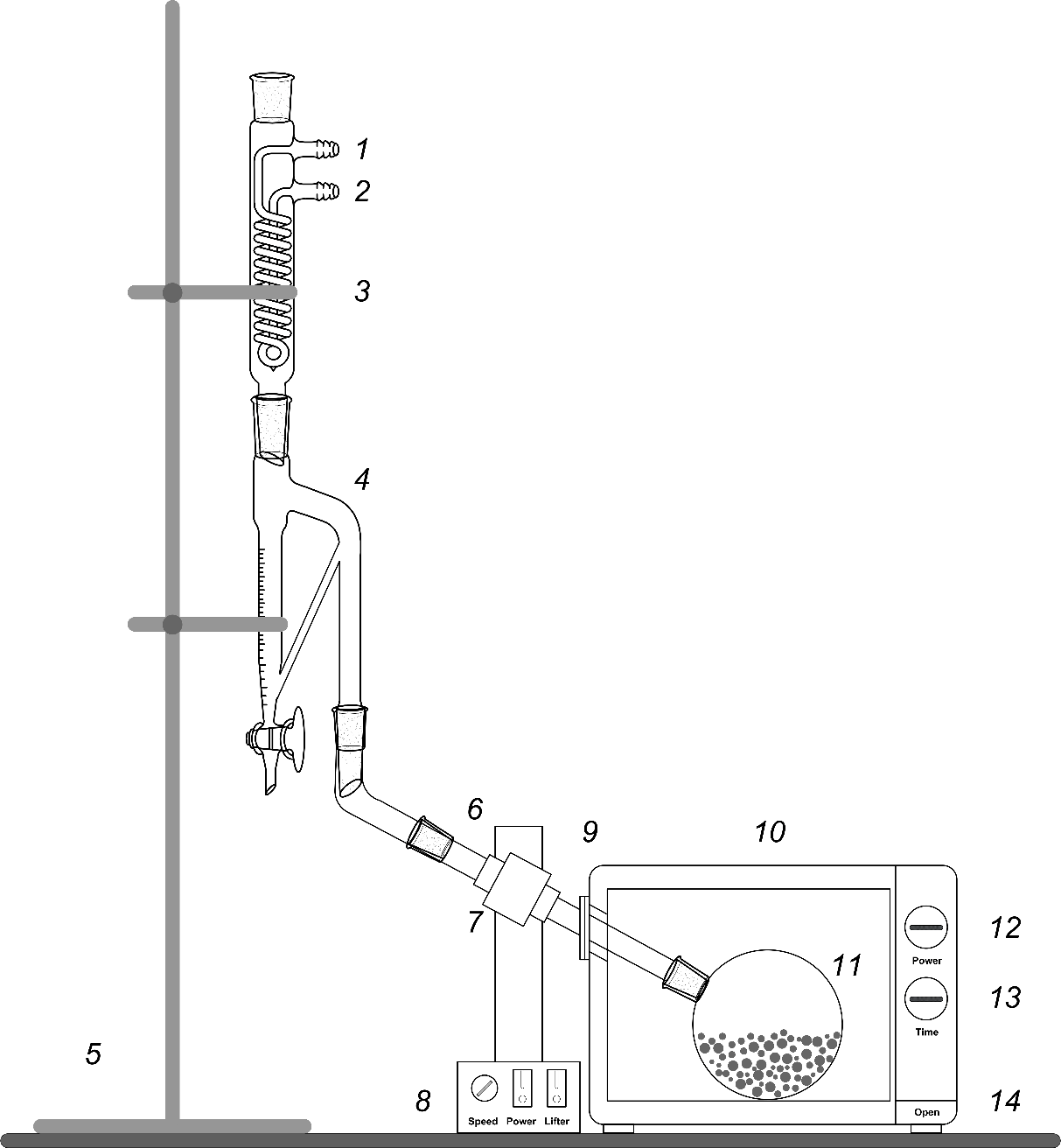


Figure S1. Schematic diagram of the salting-out solvent-free microwave mediated rotary distillation apparatus. 1. Cooling water out; 2. cooling water in; 3. reflux condenser; 4. extraction cell; 5. fixture; 6. axis of rotation; 7. rotary motor; 8. control panel; 9. anti-microwave leakage baffle; 10. microwave oven; 11. material reaction flask; 12. microwave irradiation power knob; 13. microwave irradiation time knob; 14. open knob.
